# Desmopressin for prevention of bleeding for thrombocytopenic, critically ill patients undergoing invasive procedures: A randomised, double‐blind, placebo‐controlled feasibility trial

**DOI:** 10.1002/jha2.955

**Published:** 2024-06-16

**Authors:** Michael J. R. Desborough, Emma Laing, Daphne Kounali, Ana Mora, Renate Hodge, Siobhan Martin, Helen Thomas, Cara Hudson, Joseph Parsons, Akshay Shah, Paula Hutton, Tim Parke, Matthew P. Wise, Matthew Morgan, Stuart McKechnie, Simon J. Stanworth

**Affiliations:** ^1^ Department of Clinical Haematology Oxford University Hospitals NHS Foundation Trust Oxford UK; ^2^ Radcliffe Department of Medicine University of Oxford Oxford UK; ^3^ NHS Blood and Transplant John Radcliffe Hospital Oxford UK; ^4^ NHS Blood and Transplant Clinical Trials Unit Cambridge UK; ^5^ NHS Blood and Transplant Clinical Trials Unit Bristol UK; ^6^ Department of Critical Care Oxford University Hospitals NHS Foundation Trust Oxford UK; ^7^ Nuffield Department of Clinical Neurosciences University of Oxford Oxford UK; ^8^ Department of Critical Care Royal Berkshire Hospital Reading UK; ^9^ Department of Critical Care University Hospital of Wales Cardiff UK

**Keywords:** bleeding disorders, desmopressin, thrombocytopenia

## Abstract

Thrombocytopenic patients have an increased risk of bleeding when undergoing invasive procedures. In a multicentre, phase II, blinded, randomised, controlled feasibility trial, critically ill patients with platelet count 100 × 10^9^/L or less were randomised 1:1 to intravenous desmopressin (0.3 µg/kg) or placebo before an invasive procedure. Forty‐three participants (18.8% of those eligible) were recruited, with 41 eligible for analysis. Post‐procedure bleeding occurred in one of 22 (4.5%) in the placebo arm and zero of 19 in the desmopressin arm. Despite liberal inclusion criteria, there were significant feasibility challenges recruiting patients in the critical care setting prior to invasive procedures.

## 1 BACKGROUND

Thrombocytopenia is common amongst critically ill patients, with approximately 30% having a platelet count less than 100 × 10^9^/L [1]. Thrombocytopenic patients undergoing invasive procedures have an increased risk of bleeding [2], yet the role of platelet transfusions is unclear, and there is a need to consider alternatives given there are concerns about security of supply of platelets in many countries [3].

Desmopressin is a pro‐haemostatic drug that is commonly used for patients with inherited bleeding disorders, and is under investigation for treatment of platelet dysfunction [4, 5, 6, 7]. There are currently no data evaluating the use of desmopressin in thrombocytopenic critically ill patients undergoing invasive procedures [8].

The aim of the study was to assess the feasibility of administering desmopressin or placebo to critically ill thrombocytopenic patients undergoing invasive procedures.

## 2 METHODS

We conducted a multicentre, phase II, double‐blind, randomised, controlled feasibility trial across three intensive care units (ICUs) in the United Kingdom. Ethics approval was given by South Central—Oxford C Research Ethics Committee (16/SC/0524). This report was prepared according to the CONSORT (Consolidated Standards of Reporting Trials) Extension to Pilot and Feasibility Trials guideline [9]. The trial was prospectively registered (ISRCTN12845429).

The clinical trials unit at National Health Service (NHS) Blood and Transplant managed the trial.

Inclusion criteria were: age 18 years or above; platelet count less than or equal to 100 × 10^9^/L; currently requiring care in an ICU; and due to undergo an interventional procedure such as central venous catheter (CVC) insertion. The inclusion criteria were deliberately liberal to optimise potential recruitment, and physician discretion was used to determine if patients had a bleeding risk sufficient to consider inclusion.

Exclusion criteria were haemorrhagic shock requiring resuscitation; history of ischaemic heart disease, stroke or transient ischaemic attack; patients in whom risks of fluid retention associated with desmopressin were judged clinically significant by the attending physician; traumatic brain injury or seizures; current deep vein thrombosis or pulmonary embolism; sodium less than 129 mmol/L; pregnant or breastfeeding women; congenital bleeding disorder; history of anaphylaxis to desmopressin; and thrombotic thrombocytopenic purpura.

The procedures followed were in accordance with the ethical standards of the responsible committee on human experimentation and with the *Helsinki Declaration* of 1975, as revised in 2013.

The allocation sequence was prepared using a fixed block size by an unblinded statistician independent from the trial statistician. Treatment allocation was with numbered sealed opaque envelopes. The members of the clinical team randomising participants and preparing the desmopressin/placebo were not blinded. They handed the reconstituted desmopressin/placebo to the patient's clinical team, and were not involved with the patient's trial‐related care from this point and were not allowed to reveal the treatment allocation to other staff members. Other clinical staff, the patient and outcome assessors were blinded to treatment allocation.

Participants were randomly allocated in a 1:1 ratio to either desmopressin 0.3 µg/kg in 50 mL 0.9% sodium chloride given intravenously over 20 min; or to 50 mL 0.9% sodium chloride given intravenously over 20 min. Desmopressin or placebo were administered prior to an interventional procedure (with a target of no more than 2 h before the procedure).

The primary outcome was the proportion of eligible patients meeting all inclusion criteria and none of the exclusion criteria, who were randomised to the trial and received the allocated intervention. A threshold of recruitment of 30% of eligible patients was used to define feasibility.

Clinical secondary outcomes were new onset of active bleeding up to 24 h after administration of desmopressin or placebo (measured using the HEmorrhage MEasurement [HEME] bleeding scale) [10]; exposure to blood components up to 24 h after desmopressin or placebo; thromboembolic events up to 28 days; and serious adverse events up to 28 days. Changes in measures of haemostasis were assessed as change in parameters from baseline to 30 and 120 min after infusion of desmopressin or placebo.

Confidence intervals presented are 95% and two‐sided. The statistical package SAS version 7.13 was used to conduct analyses. The 95% confidence interval for the primary outcome was based on the binomial/Clopper–Pearson exact method. Continuous variables are reported as the median and interquartile range. When measurements were censored above a known value, the Kaplan–Meier estimator was used for reporting point‐estimates for the percentiles of the empirical cumulative distribution. Discreet variables are reported as counts and %. Missing data are reported and no data were imputed. Outcomes were analysed using intention to treat or per protocol for adverse events.

## 3 RESULTS

Between 1 February 2017 and 7 June 2019, 384 patients were screened for eligibility, and 213 patients met the inclusion criteria for the trial. The most common reason for exclusion was the planned procedure taking place outside working hours of the research teams (134/171 [78%]). Forty‐three patients were randomised (two withdrew consent before starting treatment). Forty‐one patients received desmopressin/placebo (one patient was randomised in error as platelet count >100 × 10^9^/L at randomisation), with 40 receiving treatment as per protocol, representing 18.8% (95% confidence interval: 13.8%–24.7%) of eligible patients (*n* = 213). This did not meet the primary feasibility outcome. The total number of patients randomised represented 54% (43/80) of all eligible patients when excluding out‐of‐hours cases. Three participants were lost to follow‐up; one from the placebo group who was lost by Day 28 and two from the desmopressin group by Day 7 post treatment.

Patient characteristics at baseline and invasive procedures were similar between groups (Table 1). The most common invasive procedures were CVC insertions or removals. Overall adherence to the protocol, including blood sampling and timing of interventional procedures, was 25/42 (59.5%). The main barriers to adherence to the protocol were taking blood samples at the pre‐specified times before procedures and ensuring that the procedures were performed within 2 h of the desmopressin or placebo infusion.

**TABLE 1:** Baseline characteristics.

	Placebo	Desmopressin
Characteristic	*N* = 22	*N* = 21
Age (years)	60 (46–66)	58 (51–69)
Male, *N* (%)	13 (59.1)	12 (57.1)^a^
Haemoglobin (g/L)	92 (80–102)	81 (74–102)
Platelet count (×10^9^/L)	62 (47–73)	44 (36–60)
Platelet count less than 50 × 10^9^/L	6 (27)	11 (52)
Creatinine (µmol/L)	146 (63–224)	101 (72–224)
APACHE II score	27 (21–35)	26 (16–35)
Glasgow coma score	14 (3–15)	14 (3–15)
ICU admission reason, *N* (%) (National Audit and Research Centre Codes—ICNARC)		
*Infection*	15 (68.2)	11 (52.4)
*Liver cirrhosis*	2 (9.1)	1 (4.8)
*Malignancy*	–	3 (14.3)
*Bowel obstruction*	1 (4.5)	1 (4.8)
*Haemorrhage*	–	2 (9.5)
*Trauma*	2 (9.1)	–
*Other*	2 (9.1)	1 (4.8)
Renal failure		
Acute	10 (47.6)	12 (54.5)^a^
Chronic	2 (9.5)	–
Antiplatelet drugs given within 7 days of randomisation	–	1 (4.8)^a^
Anticoagulant drugs given within 7 days of randomisation	9 (40.9)	6 (28.6)^a^
Procoagulant drugs given within 7 days of randomisation	6 (27.3)	6 (28.6)
Procedures		^a^
*Central venous catheter removal*	5 (22.7)	4 (19.0)
*Vascath removal*	3 (13.6)	3 (14.3)
*Arterial line removal*	2 (9.1)	3 (14.3)
*Vascath insertion*	2 (9.1)	2 (9.5)
*Arterial line insertion*	1 (4.5)	2 (9.5)
*Pulmonary artery catheter insertion*	–	1 (4.8)
*Arterial line and central venous catheter insertion*	–	1 (4.8)
*Drain insertion*	1 (4.5)	1 (4.8)
*Drain removal*	1 (4.5)	–
*Lumbar puncture*	1 (4.5)	–
Elective procedure	20 (90.9)	20 (95.2)
Emergency procedure	2 (9.1)	1 (4.8)

*Note*: Data presented as median (interquartile range) or *n* (%).

Abbreviation: APACHE II, Acute Physiology And Chronic Health Evaluation II.

^a^ Missing data: age *n* = 1; renal failure *n* = 2; anticoagulant drugs given within 7 days of randomisation *n* = 2; procoagulant drugs given within 7 days of randomisation *n* = 2, procedure type *n* = 1.

In the 24 h after the procedure, one participant in the placebo arm (1/22, 4.5%) experienced new active bleeding (minor bleeding from a central venous catheter insertion site) compared with none in the desmopressin arm. Four of 22 (18.2%) in the placebo arm received a red cell transfusion in the 24 h after randomisation, compared with three of 21 (14.3%) in the desmopressin arm. Thrombotic events within 28 days of randomisation occurred in four of 22 (18.2%) in the placebo arm and one of 19 (5.3%) in the desmopressin arm. The proportion of patients with at least one serious adverse event was 13/22 (59.1%) in the placebo arm and 11/21 (52.4%) in the desmopressin arm (Table 2). There was one serious adverse reaction in the desmopressin arm where a transient unexpected episode of hypotension requiring increased inotrope requirements occurred during the infusion.

**TABLE 2:** Outcomes.

Safety outcomes	Placebo	Desmopressin
Bleeding up to 24 h post treatment	*N* = 22	*N* = 19^a^
New active bleeding	1 (4.5)^a^	0 (0.0)

Blood products received up to 24 h post treatment	*N* = 22	*N* = 18^b^, ^c^
Any blood component transfused	5 (25.0)	5 (27.8)
Any red cell transfusion	4 (18.2)	3 (15.8)
Any platelet transfusion	4 (18.2)	3 (15.8)
Any plasma transfusion	0	0
Any cryoprecipitate transfusion	0	0

Serious adverse events (SAE) up to Day 28	*N* = 22	*N* = 21
All SAEs	34	23
Participants with at least one SAE	13 (59.1)	11 (52.4)
Thromboembolic events^d^	4 (18.2)	1 (5.3)
Deaths	6 (27.3)	8 (38.1)

*Note*: Data presented as *n* (%).

^a^ Minor bleeding from a central venous catheter insertion site.

^b^ The participants allocated to desmopressin arm and who withdrew before the intervention are excluded.

^c^ No data available for one patient.

^d^ Thromboembolic events: In the desmopressin arm, there was one myocardial infarction (Day 1 after treatment). In the placebo arm, there was one myocardial infarction (Day 7), two deep vein thromboses (Day 9 and Day 20) and one ischaemic stroke (Day 6).

Baseline von Willebrand factor (VWF) antigen levels in both arms were significantly elevated (up to 11.3 iu/mL) compared with the normal range for VWF antigen (0.5–2.0 iu/mL). There was no difference in VWF antigen, VWF activity, VWF collagen binding activity, platelet function analyser closure time and thrombin generation parameters for those treated with desmopressin or placebo (Supporting Information).

## 4 DISCUSSION

The study successfully recruited and randomised patients to a comparison of desmopressin/placebo, but the overall proportion of potentially eligible patients recruited was low despite liberal inclusion criteria. The overall incidence of bleeding following invasive procedures in thrombocytopenic patients in our study was low, as reported elsewhere [2]. No efficacy signal was seen for desmopressin on a range of outcomes nor markers of haemostasis. We had hypothesised that increasing VWF levels would compensate for thrombocytopenia [11]. However, VWF levels did not increase, which may be due to baseline levels of VWF being very high because of acute illness [12]. It is possible that desmopressin may still exert an effect in ways that were not assessed in this study such as enhancing the formation of procoagulant platelets [13].

In the study by van Baarle et al. [2], bleeding occurred predominantly in haematology ward patients undergoing subclavian CVC insertion, and not ICU [14].

Our study indicates that the area of research prophylaxis is challenging to deliver, as also found when studying plasma prophylaxis [15]. The number of eligible patients that could be randomised and received desmopressin/placebo as a proportion of those eligible was less than the 30%, anticipated. This did not meet the primary feasibility outcome, and suggests that it would be challenging to run a definitive efficacy trial on a larger scale. If a larger, definitive trial was conducted, approaches would be needed to address consenting and the urgent nature of many of the procedures that patients undergo, many of which are out of regular working hours. Centres with 24 h per day research cover are likely to capture more patients. Additionally, inclusion of procedures with a greater bleeding risk may increase the number of participants that could be recruited.

Strengths of our study were the randomised controlled trial methodology and the inclusion of a range of patients with thrombocytopenia. The rates of concordance with the clinical administration of desmopressin or placebo were high with the exception of timing of blood tests around the procedure and ensuring the procedure took place within 2 h of administration of desmopressin or placebo.

This trial demonstrated challenges in the running of a large definitive trial evaluating time‐sensitive prophylactic interventions in critically ill thrombocytopenic patients undergoing invasive procedures.

## AUTHOR CONTRIBUTIONS

**Michael J. R. Desborough**; **Stuart McKechnie** and **Simon J. Stanworth** conceived the ideas for the trial. **Michael J. R. Desborough** wrote the first draft of the final manuscript. **Emma Laing** and **Ana Mora** managed the trial. **Daphne Kounali** did the statistical analysis with support from **Helen Thomas** and **Cara Hudson**. **Renate Hodge** and **Siobhan Martin** oversaw data management. **Akshay Shah; Paula Hutton; Tim Parke; Matthew P. Wise** and **Matthew Morgan** provided clinical expertise and led recruitment at the specialist centres in the trial. All authors had full access to all the data in the study and had final responsibility for the decision to submit for publication. **Michael J. R. Desborough** and **Daphne Kounali** have directly accessed and verified the underlying data reported in the manuscript.

## CONFLICT OF INTEREST STATEMENT

The authors declare they have no relevant conflicts of interest.

## ETHICS STATEMENT

The authors have confirmed ethical approval statement is not needed for this submission.

## PATIENT CONSENT STATEMENT

The authors have confirmed patient consent statement is not needed for this submission.

## CLINICAL TRIAL REGISTRATION

The authors have confirmed clinical trial registration is not needed for this submission.The clinical trial registration number was: ISRCTN12845429

## Supporting information

Supporting Information

## Data Availability

Individual participant data that underlie all published trial results will be available upon request from the NHS Blood and transplant clinical trials unit after de‐identification (text, tables, figures and appendices) beginning 9 months and ending 5 years following article publication. Data will be shared with investigators whose use of the data has been assessed and approved by an NHS Blood and Transplant review committee as a methodologically sound proposal. Data will be shared to achieve the aims in the approved proposal.
