# Supplementary material for: Desmopressin for prevention of bleeding for thrombocytopenic, critically ill patients undergoing invasive procedures: A randomised, double‐blind, placebo‐controlled feasibility trial
Source: EJHaem. 2024 Jun 16;5(4):772–7. doi: 10.1002/jha2.955 (PMC11327725; doi:10.1002/jha2.955)
Supplement: Supplementary file 1 — Supporting Information [file JHA2-5-772-s001.docx]

**Supplementary data**

Desmopressin for prevention of bleeding for thrombocytopenic, critically ill patients undergoing invasive procedures: a randomised, double-blind, placebo-controlled feasibility trial.

**Contents**

**List of Eligible Interventional Procedures 2**

**Consent Procedures 3**

**Randomisation procedures 6**

**Intervention Details 8**

**Assessment schedule 11**

**Laboratory Methods 13**

**Recruitment by site 13**

All laboratory outcomes 14

Summary of recruited participants 16

Feasibility outcomes 17

Figure S1. Consent process 5

Figure S2. CONSORT flow chart 16

**Table S1. List of Interventional procedures** eligible for inclusion 2

Table S2. Guidance on management of adverse events 9

Table S3. Assessment schedule 11

Table S4. Recruitment by site 13

Table S5. Laboratory outcomes 14

Table S6. Feasibility outcomes 17

**Eligible Interventional Procedures**

| - Abdominal Drain Insertion | - Liver biopsy |
| --- | --- |
| - Ascitic Drain Insertion | - Lumbar puncture |
| - Bronchoscopy | - Lung biopsy |
| - Chest Drain Insertion | - Lymph node biopsy |
| - Chest Drain Removal | - Pelvic drain insertion |
| - Colonoscopy | - Regional anaesthesia (where peri-procedural bleeding risk high) |
| - Endoscopy | - Skin or muscle biopsy |
| - Flexible Sigmoidoscopy | - Surgery |
| - Joint Aspiration or Joint Injection | - Surgical drain removal (where bleeding risk high) |
| - Kidney biopsy | - Tracheostomy insertion |
|  | - Wide-bore drain removal |
| - Line Insertions - Arterial line - Central venous catheter - Epidural catheter - PICC line - Pulmonary artery catheter - Tunnelled central line - VasCath | - Line Removals - Arterial line - Central venous catheter - Epidural catheter - PICC line - Pulmonary artery catheter - Tunnelled central line - VasCath |

**Table S1. List of Interventional procedures** eligible for inclusion

**Consent Procedures**

Three approaches were used to ensure participants’ consent for participation in this trial was given. The approach used depended on the urgency of the interventional procedure, and the availability of a suitable personal representative of the patient who is able to make a decision on their behalf. Capacity was assessed by appropriate members of the patient’s clinical team, who are trained and experienced in making assessments of mental capacity.

**If the patient was considered to have mental capacity they were invited to participate directly.** Written informed consent was obtained (if the patient was unable to read or write, then the information sheet was read to him/her and he/she was asked to mark the consent form with either a cross or a thumbprint. In this event, a full signature confirming the mark was obtained from an independent witness). If the participant lost capacity during the trial, the consent previously given when capable remained valid. If a patient with capacity refused participation in the trial and subsequently lost capacity, he/she could not be entered into the trial by seeking consent from a representative.

**If the patient lacked mental capacity, written informed consent was obtained from a representative prior to randomisation** (Medicines for Human Use (Clinical Trials) Regulations 2004). If a relative or friend of the patient was present and willing to act as the patient’s personal representative, they were approached and provided with the information sheet. This person had to be knowledgeable about the patient’s values and beliefs. The representative was given the opportunity to ask questions, and was provided with a contact point for further details about the trial.

It was preferential to approach a personal representative, however when there was no personal representative present and willing, a professional representative was approached. This was an independent doctor (not connected with the conduct of the trial) who was responsible for the care of the patient. This approach was only used when no suitable personal representative was available.

If the patient regained capacity, the trial was discussed with them and their consent obtained for continuation in the trial. This was documented in the medical records. Where consent was withheld, the patient was withdrawn from the trial.

**If the patient lacked mental capacity, the interventional procedure was required urgently and it was not reasonably practicable to seek written informed consent from a patient representative, the patient could be entered into the trial following an emergency waiver of consent** (Medicines for Human Use (Clinical Trials) Amendment No.2 Regulations 2006). The principal investigator (or other delegated Investigator) would identify an eligible patient and request agreement for entry from an independent doctor (not connected with the conduct of the trial) who was responsible for the care of the patient. Together, the principal investigator and independent doctor would ascertain whether the patient was eligible and then decide on whether or not the patient could be enrolled into the trial. This decision took into account any known views of the patient about research. If the principal investigator and independent doctor agreed, then the patient could be randomised. This decision was documented clearly in the medical records, along with the explanation of why written consent could not be obtained prior to randomisation.

For patients enrolled under this emergency consent procedure, the patient (if they regained capacity) or his/her personal or professional representative was informed about the trial as soon as it is possible. Written informed consent for trial continuation was obtained. Where consent was withheld, the patient was withdrawn from the trial.

This approach was justified for use in this trial when there was a crucial need to administer desmopressin/placebo rapidly when a decision to perform a procedure was made.

**Overview of Consent Procedures**

Is the patient eligible and due to undergo interventional procedure***** ?

**YES**

**YES**

Does patient have mental capacity?

**NO**

**NO**

**PATIENT MUST NOT BE INCLUDED IN TRIAL**

Obtain informed consent from patient.

Is a relative or friend of the patient present and willing to act as a personal representative?

**YES**

Obtain informed consent from personal representative.

**NO**

A doctor responsible for the care of the patient (and independent of the trial), should be approached.

Do they agree that the patient can be entered into the trial?

**YES**

Obtain informed consent from professional representative.

***** If the interventional procedure is required immediately and consequently there is no time to obtain prior written informed consent from the patient or patient representative – the emergency waiver of consent process can be used:

Two doctors, one independent of the trial, must agree that the patient can be randomised into the trial. Written informed consent (from the patient or patient representative) should be sought as soon as is possible after this. If consent is withheld, the participant must be withdrawn.

**Figure S1.** Consent process

**Randomisation procedures**

**Allocation – sequence generation**: The allocation sequence was prepared by an unblinded statistician independently from the trial statistician. The allocation sequence had a fixed block size.

**Allocation – concealment mechanism**: Treatment allocation was with numbered sealed opaque envelopes. These envelopes were prepared by the Independent statistician and used the randomisation schedule drawn-up by the Independent statistician. The envelopes were kept in a secure location to which the trial team had access. Each envelope was numbered and contained a card stating the randomisation number and the arm of the trial to which the participant had been allocated. Envelopes were opened in sequential numerical order.

For each participant the randomisation number was recorded on the randomisation log and the participant’s electronic case report form. The date and time of randomisation, along with the participant’s initials and date of birth were recorded on the randomisation log provided with the Sealed Envelopes, as a check that the randomisation was sequential. All envelopes, opened and unopened were collected at the end of the trial.

The trial team, research nurses, trial statistician, outcomes assessors, all members of the clinical trial unit and patients were blinded to treatment allocation. Two members of the clinical team would prepare the desmopressin or placebo prior to allocation and would hand this to the participant’s clinical team. They were not blinded to treatment allocation but took no further part in the trial. Desmopressin and placebo were non-identical at the time of preparation. After preparation, desmopressin or placebo handed to the clinical team was identical.

**Allocation – implementation**: The randomisation protocol was designed by the trial statistician. As this was a double-blind trial, the allocation sequence was generated by an independent, unblinded statistician, according to the protocol.

**Randomisation Practicalities**: The randomisation envelope was re-sealed, initialled and dated before being returned to the randomisation box for storage. The participant’s details (e.g. name, date of birth, hospital number) were written on the outside of the envelope.

The trial stock of desmopressin ampoules was stored between 2°C and 8°C, and saline vials were kept at room temperature. Fridge temperatures were required to be monitored daily and recorded in a temperature log. The Trial Manager checked logs and storage of desmopressin/placebo during monitoring visits.

**Randomisation Codes**: Each sealed envelope was numbered with a three digit randomisation number in the format Rppp, where ppp is a three digit number for the participant.

**Blinding**: The member(s) of the local team who were randomising the participants and preparing/checking the desmopressin/placebo were not blinded. These staff members received trial-specific training. They handed the reconstituted desmopressin/placebo to the patient’s clinical team and were not involved with the patient’s trial-related care from this point and were not allowed to reveal the treatment allocation to other staff members. As the duration of stability of desmopressin once suspended in 0.9% sodium chloride is unknown, it was required to be administered immediately after dilution. As a result, it had to be made on the intensive care unit prior to administration.

**Unblinding:** Only the principal investigator and delegated deputies had authority to unblind a participant and this was strongly discouraged. An unblinded treatment allocation list was also held at the site’s pharmacy department – the on-call pharmacist available 24-hours a day. No unblinding was needed.

**Intervention Details**

**Preparation of Study Drug:** Desmopressin and diluent/placebo (0.9% w/v sodium chloride) was sourced from hospital stock and labelled by the Trial Pharmacist at the participating site. The labelled stock was not blinded.

Active treatment was desmopressin, at a dose of 0.3µg/kg, diluted in 50mL 0.9% w/v sodium chloride. Placebo treatment was 50mL 0.9% w/v sodium chloride.

Once reconstituted, the infusion was labelled and handed to the patient’s clinical team for intravenous administration.

**Blinding of Study Drug:** The infusion was not administered by the same medical/nursing staff that have randomised the participant and prepared the desmopressin/placebo, and following the blinding procedure described in Appendix 3 .

**Administration of Study Drug:** The study drug was to be infused over 20 minutes (rate = 150mL/hr). Where possible, the study drug was administered through a designated line, and preferentially a central line (although the drug could be administered via a peripheral line). The infusion was not allowed to be mixed with any other medicines. It was recommended to monitor that blood pressure continuously throughout the infusion.

The gap between the infusion end time and an interventional procedure was to be between 30 and 120 minutes – but timings were recorded on the electronic case report form.

**Dose Adjustment of Study Drug:** The same dose (0.3µg/kg) of desmopressin was used for all participants in this trial.

The dose was calculated on the patient’s last recorded weight. For patients with a body mass index of greater than 30 kg/m^2^ or with fluid overload who were considered eligible for inclusion, ideal body weight was considered for the dose calculation. For patients where no weight was recorded and it was not possible to measure the weight before administration of desmopressin, ideal body weight was used. Ideal body weight was calculated using the Devine formula:

• For men: ideal body weight (kg) = 50kg + [0.91kg x (height in centimetres – 152)]

• For women: ideal body weight (kg) = 45.5kg + [0.91kg x (height in centimetres – 152)]

Guidance was given on how to manage desmopressin/placebo-related adverse events and is detailed in the Table below (Management of Adverse Events). The infusion could be stopped at any time, at the discretion of the treating clinician.

**Guidance given on Management of Adverse Events**

| **Event** | **Management** | **Estimated frequency** |
| --- | --- | --- |
| **Facial flushing (usually asymptomatic)** | If no associated hypotension and not distressing for patient, continue infusion. If unpleasant symptoms, halve infusion rate to 75 ml/hour. If no improvement stop infusion. | More than 1 in 100 |
| **Headache** | If not distressing for patient, continue infusion. If unpleasant symptoms, halve infusion rate to 75 ml/hour. If no improvement stop infusion. | More than 1 in 100 |
| **Nausea** | If not distressing for patient, continue infusion. If unpleasant symptoms, halve infusion rate to 75 ml/hour. If no improvement stop infusion. If symptoms fail to settle administer anti-emetic. | More than 1 in 100 |
| **Hypotension** | A small decrease in mean arterial pressure (approx. 10mmHg) and increase in heart rate is common following desmopressin infusion and spontaneously resolves after the infusion is complete.  In the event of a significant decrease in blood pressure (e.g. sustained reduction in systolic blood pressure >10mmHg and/or as judged significant by the bedside nurse) the rate of infusion of study drug will be reduced by half (to 75 ml/hour) and medical staff informed.  Patients on vasopressor may require a temporary small increase in vasopressor for duration of infusion if they become hypotensive.  In the event of significant hypotension (systolic blood pressure < 90mmHg, mean arterial pressure < 70mm Hg, or a systolic blood pressure decrease > 40mmHg) the study drug will be discontinued and medical staff informed. | A small decrease in mean arterial pressure (fall of less than 10mmHg) may occur in approx. 12 in 100 patients^2,3^.  Hypotension requiring a fluid bolus or increased inotrope requirements: approx. 4 in 1000 patients^2^.  *If desmopressin is given more rapidly (over 15 minutes or less) incidence of clinically significant hypotension increases significantly to approx. 9 in 100 patients^2^.* |
| **Hyponatraemia** | If significant hyponatraemia occurs then treat with fluid restriction.   - If patient is taking oral fluids then restrict to 1.5 litres max over 24 hours. - If on IV fluids then reduce maintenance fluids to 75% of standard maintenance.   Fluid boluses to treat hypotension can still be given if indicated and are not part of the fluid restriction regimen. | Any hyponatraemia  (2 in 100)  Severe hyponatraemia  (1 in 10,000) |
| **Anaphylaxis** | Stop infusion immediately and do not restart. Treat in standard way for anaphylaxis. | Data not available. Very rare. |
| **Thrombotic events (myocardial infarction, ischaemic stroke or venous thromboembolism)** | Treat in same way as for standard care. | No evidence of increased risk of thrombotic events with desmopressin infusion but this is an area of uncertainty. |

**Table S2. Guidance on management of adverse events**

| **TIMEPOINT** | **Pre-Randomisation** | **Pre-**  **Treatment** | **Treatment** | **Interventional Procedure** | **Follow-Up** | | | | |
| --- | --- | --- | --- | --- | --- | --- | --- | --- | --- |
|  |  |  |  |  | **30 minutes**  **post-treatment** | **120 minutes post-treatment** | **Day 1** | **Day 7** | **Day 28** |
| **ENROLMENT** |  |  |  |  |  |  |  |  |  |
| Eligibility Screen | **X** |  |  |  |  |  |  |  |  |
| Pregnancy Test * | **X** |  |  |  |  |  |  |  |  |
| Informed Consent^□^ | **X** |  |  |  |  |  |  |  |  |
| **INTERVENTIONS** |  |  |  |  |  |  |  |  |  |
| Administration of trial infusion |  |  | **X** |  |  |  |  |  |  |
| Interventional Procedure**^+^** |  |  |  | **X** |  |  |  |  |  |
| **ASSESSMENTS** |  |  |  |  |  |  |  |  |  |
| Full Blood Count (FBC) |  | **X** |  |  |  |  |  |  |  |
| Prothrombin Time (PT) |  | **X** |  |  |  |  |  |  |  |
| Activated Partial Thromboplastin Time (APTT) |  | **X** |  |  |  |  |  |  |  |
| Fibrinogen |  | **X** |  |  |  |  |  |  |  |
| Laboratory Assays^∆^ |  | **X** |  |  | **X** | **X** |  |  |  |
| APACHE II Assessment | **X** |  |  |  |  |  |  |  |  |
| Bleeding Assessment |  |  |  |  |  |  | **X** |  |  |
| Thromboembolic Events |  |  |  |  |  |  | **X** | **X** | **X** |
| Safety Reporting |  |  | **X** | **X** | **X** | **X** | **X** | **X** | **X** |

**Assessment schedule**

**Table S3.** Assessment schedule. * All women of childbearing potential. ^□^ Informed consent should be obtained prior to any trial procedures (see protocol section 4.3 for full details on consent procedure). If informed consent was obtained by the participant’s Legal Representative (personal or professional) – the participant should be approached for agreement to continue if they regain capacity.

**^+^** Should be performed 30-120 minutes post-administration of trial treatment. Should be performed as per standard local practice.

^∆^ Assays will be performed by the Chief Investigator. Tests to be performed: von Willebrand factor antigen, von Willebrand factor activity, von Willebrand factor collagen binding assay, platelet function analyser with adenosine diphosphate/collagen cartridge and with a P2Y cartridge, and assessment with a microfluidic flow chamber using collagen as an agonist.

**Laboratory Methods**

Changes in measures of haemostasis were assessed as change in parameters from baseline to 30 minutes and 120 minutes after infusion of desmopressin or placebo. The measures of haemostasis were VWF antigen (vWF Ag® Kit, Siemens, Marburg, Germany), VWF activity GP1bM (Innovance® VWF Ac kit, Siemens, Marburg, Germany), VWF collagen binding activity (ZYMUTEST vWF:CBA ELISA, Hyphen BioMed, Epsom, Surrey), platelet function analyser 200 with collagen/ADP (C-ADP cartridge, Innovance, Siemens Healthcare Diagnostics GmBH) or P2Y cartridge (Innovance PFA P2Y cartridge; Siemens Healthcare Diagnostics GmBH), and thrombin generation parameters (lag time, time to peak, endogenous thrombin potential and peak thrombin generation). Thrombin generation was assessed using calibrated automated thrombinography. A combination of either 1 pM or 5 pM recombinant human tissue factor (TF), and 4 μM phospholipid were used as activators with the coagulation reaction triggered by the addition of calcium chloride which was added in combination with Z-Gly-Gly-Arg-amino-methyl-coumarin, a thrombin-sensitive fluorogenic substrate (all reagents from Thrombinoscope BV, Maastricht, The Netherlands). Analysis was performed using a Fluoroskan Ascent FL microplate fluorometer and luminometer (Thermo Scientific, Helsinki, Finland) and Thrombinoscope version 5 software (Thrombinoscope, BV, Maastricht, The Netherlands).

**Recruitment by site**

|  | **Centre** | | |  |
| --- | --- | --- | --- | --- |
|  | **Oxford University Hospitals, Oxford** | **Royal Berkshire Hospital, Reading** | **University Hospital of Wales, Cardiff** | **Total** |
| Screening start date | 1 February 2017 | 28 February 2018 | 3 September 2018 |  |
| Screening end date | 7 June 2019 | 7 June 2019 | 7 June 2019 |  |
| Number of patients screened | 280 | 48 | 56 | **384** |
| Number of eligible patients | 167 | 20 | 26 | **213** |
| Number consented | 17 | 12 | 14 | **42** |
| Number of randomisations  Placebo Desmopressin | 17  9  8 | 12  6  6 | 14  7  7 | **43**  **22**  **21** |
| Recruitment per month | 0.61 | 0.75 | 1.56 | **0.81** |

**Table S4. Recruitment by site.**

| **Research Assay** | **Testing Period** | **Placebo** | **Desmopressin** |
| --- | --- | --- | --- |
| VWF antigen (IU/mL) | Pre-treatment | 4.5 (3.8-7.4); n=22 | 4.8 (4.2-7.5); n=19 |
|  | 30 min Post-treatment | 4.7 (4.2-7.3); n=21 | 4.6 (4.2-7.7); n=18 |
| VWF activity (IU/mL) | Pre-treatment | 4.8 (3.7-5.6); n=21 | 4.9 (3.8-7.4); n=18 |
|  | 30 min Post-treatment | 4.8 (3.9-6); n=20 | 4.8 (4.1-6.1); n=17 |
| VWF collagen binding (IU/mL) | Pre-treatment | 4.4 (3.1-8.2); n=17 | 6 (4.1-8.7); n=16 |
|  | 30 min Post-treatment | 4.7 (3.1-8.9); n=16 | 6.3 (4-7.9); n=14 |
| PFA-200 closure time with ADP/collagen cartridge (seconds) | Pre-treatment | 184 (149->300); n=10 | >300 (182->300); n=7 |
|  | 30 min Post-treatment | >300 (166->300); n=10 | >300 (174->300); n=6 |
| PFA-200 closure time with P2Y cartridge (seconds) | Pre-treatment | 248 (135->300); n=10 | >300 (139->300); n=7 |
|  | 30 min Post-treatment | 137 (90-186); n=10 | 145 (83->300); n=6 |
| Thrombin generation peak (nM) with 1 pM TF | Pre-treatment | 178 (172-263); n=9 | 183.5 (99-292); n=6 |
|  | 30 min Post-treatment | 202 (153.5-248.5); n=8 | 182 (87-376); n=5 |
| Thrombin generation peak (nM) with 5 pM TF | Pre-treatment | 207 (183-304); n=9 | 255.5 (159-335); n=6 |
|  | 30 min Post-treatment | 220 (187.5-282); n=8 | 228 (177-331); n=5 |
| Thrombin generation time to peak with 1 pM TF (minutes) | Pre-treatment | 11.2 (9.8-15.2); n=9 | 13.6 (7.7-19.9); n=6 |
|  | 30 min Post-treatment | 11.4 (9.2-12.6); n=8 | 12.7 (6-18.8); n=5 |
| Thrombin generation time to peak with 5 pM TF (minutes) | Pre-treatment | 6.4 (5.1-6.8); n=9 | 6.3 (4.7-9.8); n=6 |
|  | 30 min Post-treatment | 6.2 (5.5-7.9); n=8 | 7.6 (4.3-9.7); n=5 |
| Thrombin generation time to peak with 1 pM TF (minutes) | Pre-treatment | 11.2 (9.8-15.2); n=9 | 13.6 (7.7-19.9); n=6 |
|  | 30 min Post-treatment | 11.4 (9.2-12.6); n=8 | 12.7 (6-18.8); n=5 |
| Thrombin generation time to peak with 5 pM TF (minutes) | Pre-treatment | 6.4 (5.1-6.8); n=9 | 6.3 (4.7-9.8); n=6 |
|  | 30 min Post-treatment | 6.2 (5.5-7.9); n=8 | 7.6 (4.3-9.7); n=5 |
| Thrombin generation ETP  (nM-min) with 1 pM TF | Pre-treatment | 1594 (1488-1675); n=9 | 1434.5 (814-1998); n=6 |
|  | 30 min Post-treatment | 1595 (1221.5-1670); n=8 | 1303 (915-1883); n=5 |
| Thrombin generation ETP  (nM-min) with 5 pM TF | Pre-treatment | 1513 (1282-1832); n=9 | 1535 (1094-2164); n=6 |
|  | 30 min Post-treatment | 1792 (1351.5-2089); n=8 | 1461 (1418-1692); n=5 |
| Thrombin generation lag time with 1 pM TF (minutes) | Pre-treatment | 8.7 (6.8-11.5); n=9 | 9.9 (5-16.1); n=6 |
|  | 30 min Post-treatment | 8.4 (7.7-9.5); n=8 | 10 (4-14.7); n=5 |
| Thrombin generation lag time with 5 pM TF (minutes) | Pre-treatment | 3.4 (2.9-3.9); n=9 | 3.5 (2.7-6.8); n=6 |
|  | 30 min Post-treatment | 3.4 (3-4.9); n=8 | 4.3 (2.2-6.3); n=5 |

**Table S5.** Laboratory outcomes. ADP – adenosine diphosphate, ETP – endogenous thrombin potential, PFA – platelet function analyser, TF – tissue factor, VWF – Von Willebrand Factor. VWF assays were performed at three sites. PFA was performed at two sites and thrombin generation was performed at one trial site.

**Summary of recruited participants**

Eligible n=214

Excluded n=171

- Not approached n=5
- Declined to participate n=15
- Clinical decision n=8
- Patient missed n=134
- Other n=9

Bleeding and thrombosis assessment: Day 1 n=20

*-Lost due to death n=2*

Thrombosis assessment: Day 7 n=20

Thrombosis assessment Day 28 n=15

*-Lost due to death n=4*

*-Lost to follow-up n=1 (Discharged home)*

Available for analyses **Placebo**: n=22

- *Feasibility: Protocol Adherence*
- *Feasibility: Time taken to administer treatment*
- *Efficacy: Research Assays*

Allocated to receive **Placebo** n=22

- Received allocated intervention n=22

*-Randomised in error: not eligible n=1*

- Did not receive allocated intervention: n=0

Available for analyses **Desmopressin** n=21

- *Feasibility: Protocol Adherence*
- *Feasibility: Time taken to administer treatment*
- *Efficacy: Research Assays*

Allocated to receive **Desmopressin** n=21

- Received allocated intervention n=19
- Did not receive allocated intervention n=2

-*Withdrawn n=2*

Bleeding and thrombosis assessment^:^ Day 1 n=18

*-Lost to follow-up n=1 (Discharged home)*

Thrombosis assessment: Day 7 n=11

*-Lost due to death n=6; Lost to follow-up n=1*

Thrombosis assessment: Day 28 n=9

*- Lost due to death n=2*

## Allocation

## Allocation

## Allocation

## Allocation

## Allocation

## Allocation

## Allocation

## Allocation

## Allocation

## Allocation

## Allocation

## Allocation

## Allocation

## Allocation

## Allocation

## Allocation

## Allocation

## Allocation

## Allocation

## Allocation

## Allocation

## Allocation

## Allocation

## Allocation

## Allocation

## Allocation

## Allocation

## Allocation

## Allocation

## Allocation

## Allocation

## Allocation

## Follow-up

## Follow-up

## Follow-up

## Follow-up

## Follow-up

## Follow-up

## Follow-up

## Follow-up

## Follow-up

## Follow-up

## Follow-up

## Follow-up

## Follow-up

## Follow-up

## Follow-up

## Follow-up

## Follow-up

## Follow-up

## Follow-up

## Follow-up

## Follow-up

## Follow-up

## Follow-up

## Follow-up

## Follow-up

## Follow-up

## Follow-up

## Follow-up

## Follow-up

## Follow-up

## Follow-up

## Follow-up

## Post-treatment assessments

## Post-treatment assessments

## Post-treatment assessments

## Post-treatment assessments

## Post-treatment assessments

## Post-treatment assessments

## Post-treatment assessments

## Post-treatment assessments

## Post-treatment assessments

## Post-treatment assessments

## Post-treatment assessments

## Post-treatment assessments

## Post-treatment assessments

## Post-treatment assessments

## Post-treatment assessments

## Post-treatment assessments

## Post-treatment assessments

## Post-treatment assessments

## Post-treatment assessments

## Post-treatment assessments

## Post-treatment assessments

## Post-treatment assessments

## Post-treatment assessments

## Post-treatment assessments

## Post-treatment assessments

## Post-treatment assessments

## Post-treatment assessments

## Post-treatment assessments

## Post-treatment assessments

## Post-treatment assessments

## Post-treatment assessments

## Post-treatment assessments

Randomised n=43

Randomized (n=43 )

Randomized (n=43 )

Randomized (n=43 )

Randomized (n=43 )

Randomized (n=43 )

Randomized (n=43 )

Randomized (n=43 )

Randomized (n=43 )

Randomized (n=43 )

Randomized (n=43 )

Randomized (n=43 )

Randomized (n=43 )

Randomized (n=43 )

Randomized (n=43 )

Randomized (n=43 )

Randomized (n=43 )

Randomized (n=43 )

Randomized (n=43 )

Randomized (n=43 )

Randomized (n=43 )

Randomized (n=43 )

Randomized (n=43 )

Randomized (n=43 )

Randomized (n=43 )

Randomized (n=43 )

Randomized (n=43 )

Randomized (n=43 )

Randomized (n=43 )

Randomized (n=43 )

Randomized (n=43 )

Randomized (n=43 )

## Enrolment

## Enrollment

## Enrollment

## Enrollment

## Enrollment

## Enrollment

## Enrollment

## Enrollment

## Enrollment

## Enrollment

## Enrollment

## Enrollment

## Enrollment

## Enrollment

## Enrollment

## Enrollment

## Enrollment

## Enrollment

## Enrollment

## Enrollment

## Enrollment

## Enrollment

## Enrollment

## Enrollment

## Enrollment

## Enrollment

## Enrollment

## Enrollment

## Enrollment

## Enrollment

## Enrollment

## Enrollment

Screened for eligibility n=384

Screened for eligibility (n=384^¥^ )

Excluded n=170

- History of ischaemic heart disease, stroke or transient ischaemic attack n=39
- Traumatic brain injury and/or seizures n=27
- Haemorrhagic shock n=24
- Hyponatraemia n=23
- Venous thromboembolism n=12
- Risk of fluid retention n=13
- Insufficient understanding of English n=6
- Thrombotic thrombocytopenic purpura n=8
- Other n=18

## Screened

Randomised & received allocated treatment n= 41

**Figure S2. CONSORT flow diagram**

**Feasibility outcomes**

|  | Placebo  (N=21) | Desmopressin  (N=21) |
| --- | --- | --- |
| IMP administered as allocated | 21 (100.0) | 19 (90.5) |
| IMP infusion started | 21 (100.0) | 19 (90.5) |
| IMP infusion completed (not stopped early) | 21 (100.0) | 18 (85.7) |
| Pre-treatment blood sample taken ≤120 minutes before start of IMP infusion | 20 (95.2) | 18 (85.7) |
| 30 minutes post-treatment blood sample taken | 21 (100.0) | 17 (81.0) |
| Interventional procedure carried out between 30 and 120 minutes after end of IMP infusion | 19 (90.5) | 15 (71.4) |
| 120 minutes post-treatment blood sample taken | 14 (66.7) | 14 (66.7) |
| Overall adherence to protocol | 14 (66.7) | 11 (52.4) |
| Time from randomisation to start of IMP (min) | 18 (10-29) | 19 (10-30) |

**Table S6.** Feasibility outcomes. IMP – investigational medicinal product (desmopressin or placebo)
